# From Waste to Functional Feed Ingredient: Biochemical and SHK-1 Cell Line Evaluation of Black Soldier Fly Larvae for Aquaculture Nutrition

**DOI:** 10.3390/antiox14101172

**Published:** 2025-09-26

**Authors:** Julio Camperio, Jorge Parodi, Pamela Olivares-Ferretti, Jorge A. Suarez, Daniel D. Benetti

**Affiliations:** 1 Rosenstiel School of Marine, Atmospheric, and Earth Science, University of Miami, Miami, FL 33136, USA; dbenetti@earth.miami.edu; 2 Sociedad Laboratorio, Investigación y Educación Chávez-Parodi Limitada, Tonalli Ltda, Temuco 4780000, Chile; jorge.parodi@uautonoma.cl (J.P.); ps.olivares.ferretti@gmail.com (P.O.-F.); 3 Departamento de Análisis de Datos, Facultad de Ciencias Sociales, Universidad Autónoma de Chile, Temuco 4780000, Chile

**Keywords:** Black Soldier Fly Larvae Meal, functional feed ingredient, SHK-1 cell lines, oxidative stress, aquaculture nutrition

## Abstract

Black Soldier Fly Larvae Meal (BSFLM) has gained attention as a sustainable feed ingredient in aquaculture, yet its functional properties at the cellular level remain underexplored. This study evaluated the antioxidative and proliferative effects of BSFLM derived from larvae fed different waste-based substrates (Kitchen Waste (KW); Agricultural Waste (AW); Aquaculture Sludge (AS); Aquaculture Offal (AO); Mix (MX)), using the Atlantic salmon (*Salmo salar*) SHK-1 cell line as an in vitro model. BSFLM treatments were assessed through four assays: oxidative stress mitigation under H_2_O_2_ exposure, baseline cellular proliferation, proliferation under protein-standardized conditions, and recovery from serum starvation. Each assay was carried out in three independent experiments with three replicates per treatment, and changes in coloration were quantified using MTT (3-(4,5-dimethylthiazol-2-yl)-2,5-diphenyltetrazolium bromide). The results showed that BSFLM from plant-based substrates, particularly MX and KW diets, significantly (*p* < 0.05) improved cell viability across all assays. Under oxidative stress, MX (121.1% ± 5.9) and AW (119.9% ± 6.1) treatments maintained viability levels comparable to Vitamin C (119.3% ± 3.8) (250 ppm of DSM Stay-C) and the control (137.5% ± 11.6). In proliferation assays, MX (207.6% ± 16.3) and KW (196.3% ± 11.1) outperformed animal-based treatments AO (122.6% ± 4.4) and AS (113.1% ± 3.7), and these effects persisted under protein-standardized conditions, although the statistical significance was reduced. In the recovery from serum starvation assay, cells treated with MX (45.5% ± 1.9) and KW (42.0% ± 0.4) exhibited markedly higher viability than AS (15.5% ± 1.9) and AO (14.8% ± 2.2). The biochemical composition of BSFL reared on different substrates, including proximate, amino acid, fatty acid, and polyphenol profiles, was analyzed to contextualize the observed cellular responses. These findings highlight the superior functional properties of BSFLM derived from plant-based substrates and support its potential use as a targeted functional feed ingredient in aquaculture feed formulations.

## 1. Introduction

Black Soldier Fly Meal (BSFLM) has emerged as a novel ingredient in aquaculture, offering a sustainable alternative to traditional ingredients. However, due to its current cost constraints, adequate nutritional profile, and bioactive functional properties (modulating intestinal microbiota, enhancing disease resistance, and supporting immune responses), BSFLM might be better positioned as a functional ingredient rather than a bulk protein source in aquafeeds. These functional benefits necessitate a deeper understanding of their cellular and physiological impacts before widespread application.

BSFL offer a biotechnological solution for converting organic waste into high-value feed ingredients that would otherwise be transformed into low-value by-products or disposed of. Once fed with nutritionally adequate feeds, the fresh larvae can be transformed into a meal and oil, with a frass (excrement and other waste) as a by-product. A significant proportion of BSFL research in aquaculture is centered around in vivo nutritional studies were graded levels of traditional ingredients are replaced with the insect meal. While this knowledge base is essential in determining optimal dietary inclusions whilst achieving desirable growth and health performance, little is known about the cellular effects of BSFL meal on cell lines of commercially important fish species, a research gap that the present novel investigation attempts to fill.

Through in vivo trials with species such as sea bream, meager, and salmon, BSFLM has demonstrated additional benefits beyond its nutritional profile and fishmeal replacement, indicating improvements in gut health and microbial diversity, nutrient digestibility, and immune responses [1,2,3]. In fish such as rainbow trout, gilthead seabream, and striped catfish the insect oil is a suitable replacement for fish oil without negatively affecting growth performance, whole-body composition, nutrient retention, and digestibility [2,4,5]. On the other hand, the larval frass which has been originally treated as an agricultural fertilizer, has also recently been investigated as a promising aquaculture ingredient for omnivorous species such as shrimp and catfish [6,7,8]. An advantage of adopting in vitro cell lines as a complement or precursor to in vivo trials is the creation of valuable mechanistic insights into basic cellular effects of additives and ingredients, access to a tool for rapid screening and evaluation of feed components, and the reduction of animal use and infrastructure cost related to live-animal husbandry [9]. Beyond the nutritional effects on cells, this biotechnology can be used for toxicological, pathological, and immunological studies, greatly increasing its applications to different sectors of the aquaculture industry [10].

The nutritional profile of BSFLM can be substantially modulated by the composition of the substrates consumed by the larvae. This applies not only to its macronutrient content (protein, lipid, and ash) but also to its amino acid and fatty acid profiles, as well as to its total polyphenol concentration (TPC) [11,12,13,14]. It must be noted that this nutritional plasticity is not manifested only in BSFL, but research has shown that protein and lipid content of crickets (*Gryllus bimaculatus*) and mealworms (*Tenebrio molitor*) can also be manipulated through the selection of specific substrates [15,16]. Diets rich in protein and fiber generally result in larvae with elevated protein content, whereas fruit- and vegetable-based substrates tend to produce larvae with comparatively lower protein levels [17]. Fatty acid composition in BSFL reflects dietary inputs. For example, supplementing larval diets with fish oil or linseed oil has been shown to increase omega-3 fatty acids, such as eicosapentaenoic acid (EPA) and docosahexaenoic acid (DHA), within the larval biomass [11,12]. Similarly, the inclusion of fish offal in larval diets significantly enhances omega-3 content, highlighting the potential of BSFLM as a partial substitute for fish oil in aquafeeds [11,12,14]. The ash content of BSFL biomass is closely correlated with the mineral composition of the feed substrate. Studies indicate a near-linear relationship between substrate ash content and larval ash deposition, which is often considered an anti-nutritional factor [18]. Beyond macronutrients, BSFL are capable of assimilating bioactive compounds from agri-food by-products. Substrates such as olive leaves, olive pomace, quinoa husk, tomatoes, lettuce, dragon fruit, sweet potatoes, yuca, and taro have been shown to enhance the incorporation of polyphenols into larval tissue [14,19]. These compounds are of particular interest because they may contribute antioxidative and health-promoting properties to animal diets. While proteins and lipids remain central to supporting growth and metabolic processes, feeding BSFL with substrates enriched in bioactive compounds creates the opportunity to deliver functional, health-enhancing ingredients [20]. In particular, polyphenol-enriched BSFLM could be evaluated for its antioxidative potential in aquaculture systems. In vitro approaches, such as assays using aquaculture-relevant cell lines, provide a promising tool for testing the cellular antioxidative and proliferative effects of polyphenol-enriched BSFLM. Despite the growing use of BSFLM in in vivo feeding trials, its functional effects at the cellular level remain largely unexplored.

Immortalized human cell lines (HEpG2, Caco-2, and HeLa) are invaluable tools that allow for the in vitro investigation of phytochemicals (apricot kernel, wild peach, and plumwood) on cellular antioxidation and proliferation, demonstrating the potential for prevention and treatment of chronic health disorders [21,22]. In terms of aquaculture, cell lines and in vitro models provide insights into the cellular effects of additives, ingredients, and pharmaceuticals. For example, the SHK-1 cell line, derived from Atlantic salmon head kidney, has been extensively used to study immunomodulation, oxidative stress, and cellular responses to dietary components [23,24,25,26]. Studies have demonstrated its utility in evaluating the protective effects of silymarin against oxidative stress and its role in modulating immune responses [24,26]. Additionally, SHK-1 cells have been employed to assess the impact of micronutrients such as selenium in enhancing resistance to intracellular pathogens, providing critical insights into host–pathogen dynamics [25].

The SHK-1 cell line was selected for this study because it originates from the head kidney of Atlantic salmon (*Salmo salar*), which is a central immune organ responsible for hematopoiesis and regulation of both innate and adaptive immune responses [27]. This makes SHK-1 particularly suitable for assessing cellular proliferation, oxidative stress, and cytoprotective mechanisms, processes highly relevant to evaluating functional feed additives in aquaculture [26]. Alternative salmonid cell lines exist, such as intestinal epithelial RTgutGC models that are useful for studying nutrient absorption, barrier integrity, and interactions at the gut interface, or gill cell lines such as RTgill-W1 that are valuable for examining osmoregulation and mucosal immunity [28,29]. However, these models do not directly reflect the systemic immune and stress-related pathways that are mediated by head kidney leukocytes. By contrast, SHK-1 provides a robust, scalable in vitro platform that captures both immune and stress physiology, enabling mechanistic screening of dietary ingredients prior to in vivo validation [24,26].

This study represents the first investigation into the cellular effects of BSFL meal using the SHK-1 salmonid cell line. We hypothesized that BSFLM derived from different substrates would differentially modulate its functional properties, particularly antioxidant potential, reflecting substrate-driven variation reported in previous studies [14]. In line with this, we further hypothesized that BSFLM could enhance antioxidative activity, influence SHK-1 cell proliferation under nutrient-rich and nutrient-deprived conditions and exert measurable effects even when standardized to an equal protein dose. By leveraging the SHK-1 model, this research provides foundational evidence for the bioactive properties of BSFLM, supporting its potential application as a sustainable aquafeed functional ingredient and laying the groundwork for future in vitro and in vivo validation.

## 2. Materials and Methods

### 2.1. Black Soldier Fly Larvae, Nutritional Composition, and Total Polyphenol Concentration

**Figure A1:**
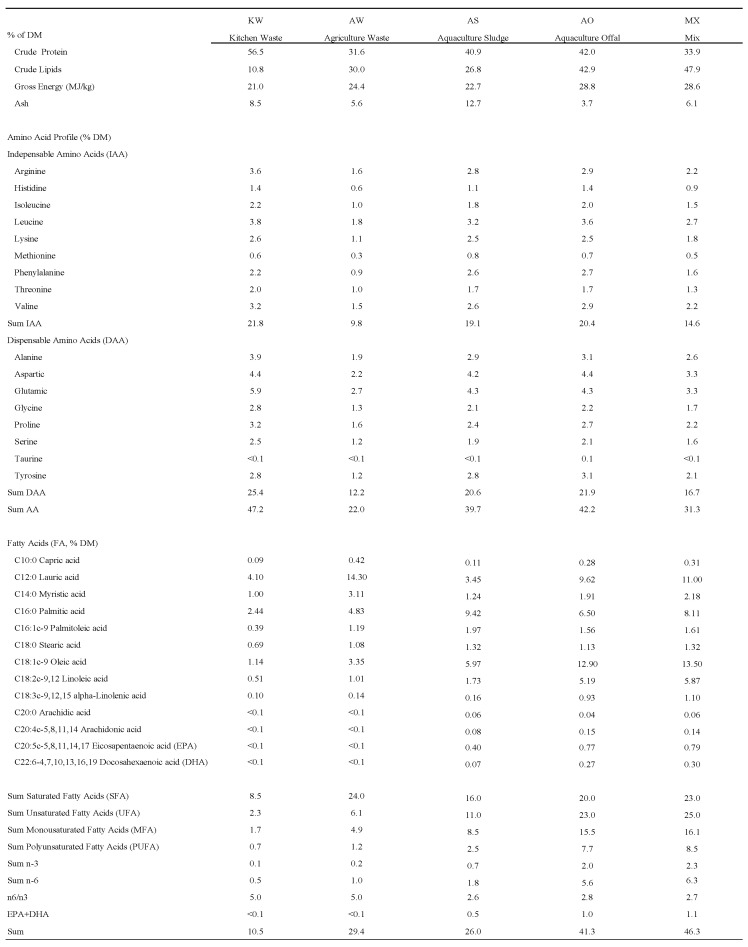
Proximate, amino acid, and fatty acid composition, as well as the polyphenol content, of the larvae after consuming their respective feed for 15 days. Kitchen Waste (KW, 50/50 romaine lettuce and tomatoes), Agricultural Waste (AW, 25/25/25/25 dragon fruit, sweet potatoes, yuca, and taro), Aquaculture Sludge (AS, waste sludge from a salmon RAS), Aquaculture Offal (AO, salmon offal post-processing), and Mix (MX, 25/25/25/25 of KW, AW, AS, and AO). Data sourced from Camperio et al., 2025 [14].

The detailed rearing protocol of the BSFL fed with the five distinct feed sources, along with analyses of nutritional composition and TPC are comprehensively described in Camperio et al. (2025) (Figure A1 in Appendix A) but generally encompasses the following [14]. Five feeds were prepared using organic waste from various commercial sectors: Kitchen Waste (KW; 50/50 lettuce and tomatoes), Agricultural Waste (AW; equal parts dragon fruit, sweet potato, yuca, and taro), Aquaculture Sludge (AS; salmon RAS sludge), Aquaculture Offal (AO; post-processing salmon offal), and a Mix (MX; equal parts KW, AW, AS, AO). All feeds were chopped fresh and stored frozen at −4 °C, then thawed for 12 h before use. BSFL (5-day old) were obtained from Symton Black Soldier Fly (College Station, Texas) and reared in containers with 200 larvae, 60 g of Hydrated Coconut Coir (HCC), and feed equal to 500% of larval biomass. Conditions were 27.1 ± 0.2 °C, 76.4 ± 0.3% humidity, and constant darkness (24:0 D:L). Each of the five treatments had four replicates. Biomass was recorded and substrate was replaced every 3 days until Day 15, when the trial concluded. Larvae and HCC were separated using a 500 µm sieve. Larvae and feed samples were dried at 105 °C for 24 h, cooled in a desiccation chamber overnight, ground into a powder using a ceramic pestle and mortar, vacuum-packed, and analyzed by a NATA-accredited lab. Protein was measured via combustion and nitrogen quantification (×6.25). Fat was determined using the Folch method with chloroform/methanol extraction. Energy was assessed using a Parr 6200 bomb calorimeter (Moline, IL, USA). Amino acids were quantified by HCl hydrolysis followed by LC-PDA-MS, and fatty acids were analyzed by gas chromatography of methyl esters. Total polyphenols in BSFL were analyzed using the Folin–Ciocalteu method. Extracts were reacted with diluted Folin reagent and sodium carbonate, incubated for 30 min, and absorbance was measured at 765 nm. Results were expressed as mg gallic acid equivalents (GAE) per 100 g, based on a gallic acid standard curve. All measurements were performed in quadruplicate.

### 2.2. Cellular Culture

Head kidney-derived cells from *Salmo salar* (SHK-1; obtained from ATCC, Manassas, VA, USA.) were maintained and cultured at 17 °C in sterile T-25 flasks containing DMEM/F12 with GlutaMAX (Gibco) supplemented with 10% fetal bovine serum (FBS; Gibco) and 1× antibiotic-antimycotic solution (Gibco). Cells were used for all experiments at passage 10. All cell manipulations and subculturing procedures were conducted in a biosafety cabinet (Biobase BBS-V500, Jinan, Shandong, China) under UV sterilization and constant HEPA-filtered airflow, as similarity described by Sanchez et al. (2016), Olivares-Ferretti et al. (2016), Olivares-Ferretti et al. (2020), and Olivares-Ferretti et al. (2024) [24,26,30,31]. The different experimental BSFLM were first ground into a fine powder using a ceramic pestle and mortar and then solubilized in complete culture media in 15 mL tubes. The 15 mL tubes were then placed on a vortex for 1 min, after which they were allowed to sit undisturbed for 30 min. After the resting period only the supernatant was filter-sterilized (0.22 µm) and utilized for the cell lines assays, while the insoluble and precipitated fraction was disposed of. Each assay (antioxidant, proliferation, protein standardization, and starvation) was performed as three independent biological replicates on separate days using fresh cell preparations, fresh culture medium, and freshly prepared reagents. Within each biological replicate, each treatment was plated in triplicate technical wells on a 96-well plate. Technical triplicates were averaged within each biological replicate, and those per-replicate means were used for all statistical analyses. Cell viability was measured with the MTT assay kit (Merck, Darmstadt, Germany).

### 2.3. MTT Assays

The MTT assay is a colorimetric method for assessing cell viability and proliferation by measuring mitochondrial activity. Viable cells reduce the yellow tetrazolium salt (MTT) to purple formazan crystals, which are then solubilized and quantified spectrophotometrically, with absorbance directly proportional to the number of living cells. For this study, cell viability was evaluated using a commercial MTT assay kit (Merck) following the manufacturer’s instructions. Briefly, SHK-1 cells were seeded into 96-well plates and allowed to adhere overnight. After experimental treatments, 10 µL of MTT solution was added to each well containing 100 µL of culture medium and incubated for 4 h at 21 °C under standard culture conditions. Following incubation, 100 µL of solubilization buffer (10% SDS in 0.01 M HCl) was added directly to each well, and the plates were incubated overnight at 21 °C in a humidified atmosphere to ensure complete dissolution of the formazan crystals. Absorbance was then measured at 520–600 nm with a reference wavelength > 650 nm using a microplate reader (Biobase BK-L10D, Jinan, Shandong, China). Cell viability was expressed as the percentage of absorbance relative to untreated control cells.

### 2.4. Cellular Antioxidation

Cells were seeded into 96-well plates at a density of 1 × 10^6^ cells/mL in 200 µL of complete culture medium containing 10% FBS. After 24 h, cells were treated with experimental BSFLM at a concentration of 1.4% in complete culture medium and exposed concurrently to 0.1 µM H_2_O_2_ for 12 h. Preliminary tests dissolved BSFLM in culture medium without saturation to keep proteins and oligopeptides soluble. A log-spaced dose–response (0.14–14%) identified 1.4% as a mid-range cellular response and non-saturating working dose. Control wells received complete culture medium only, while negative controls were exposed to 0.1 µM H_2_O_2_ in medium without BSFLM, previously identified as the LC50 concentration. Cellular viability was quantified using the MTT assay kit (Merck) described above. Data are presented as a percentage viability relative to the untreated control (complete medium + 10% FBS). The cellular antioxidation assay was carried out in three independent experiments, each having three replicates per treatment.

### 2.5. Cellular Proliferation

Cells were seeded into 96-well plates at a density of 3 × 10^5^ cells/mL in 200 µL of complete culture medium supplemented with 10% FBS. After 24 h, cells were exposed to BSFLM at 1.4% concentration in complete culture medium and incubated for 5 days. This time interval was selected due to previous studies by Olivares et al. 2025 and Sanchez et al., 2016 [24,26] who indicated that these SHK-1 in vitro cell models require 5 days to reach confluence. Cellular viability was determined by MTT assay (Merck) as described above. Results are expressed as percentage viability relative to the untreated control (complete medium + 10% FBS). The cellular proliferation assay was carried out in three independent experiments, each having three replicates per treatment.

### 2.6. Protein Standardization

BSFL meal samples were sent to an accredited laboratory for crude protein (CP) analysis, and treatment-specific CP values are listed in Figure A1 in Appendix A. Using those CP values, dosing suspensions were prepared so that the final protein concentration in culture medium was 1.4% (*w*/*v*; 14 mg protein/mL) for every treatment. For each meal, the calculated mass of powder was dispersed in serum-free medium and vortexed to homogeneity, then 0.22 µm filtered and diluted with complete medium (10% FBS) to reach 1.4% protein. All wells received the same total volume and the same final FBS concentration (10%). A medium-only control was prepared identically but without meal (vortexed, filtered, and diluted as above). Cellular viability was determined by MTT assay (Merck) as described above. Results are expressed as percentage viability relative to the untreated control (complete medium + 10% FBS). The cellular proliferation assay was carried out in three independent experiments, each having three replicates per treatment.

### 2.7. Starvation

The starvation assay comprised two trials. The first trial aimed to identify the minimal concentration of FBS capable of significantly enhancing cell viability following starvation. Cells were seeded in 96-well plates at a density of 1 × 10^5^ cells/mL. The control group received complete culture medium with 10% FBS; the negative control received complete medium without FBS, and treatment groups received incremental FBS concentrations (0.001%, 0.1%, 1%, 10%, and 20%). The second trial included control (complete medium + 10% FBS), negative control (complete medium without FBS), a treatment receiving complete medium with 1% FBS, and additional treatments containing 1.4% of the different BSFLM combined with 1% FBS. After 24 h of incubation, cell viability was determined by MTT assay (Merck) as described above. All values are presented as a percentage viability relative to the control (complete medium + 10% FBS). The starvation was carried out in three independent experiments, each having three replicates per treatment.

### 2.8. Statistical Analysis

Statistical analysis was conducted using GraphPad Prism software (version 8.0.1). Experimental data were analyzed by one-way analysis of variance (ANOVA), followed by Tukey’s Honest Significant Difference (HSD) post hoc test to identify significant differences among treatment means. Statistical significance was set at *p* < 0.05. Data are presented as mean ± standard error of the mean (SEM).

## 3. Results

### 3.1. Cellular Antioxidation

**Figure 1:**
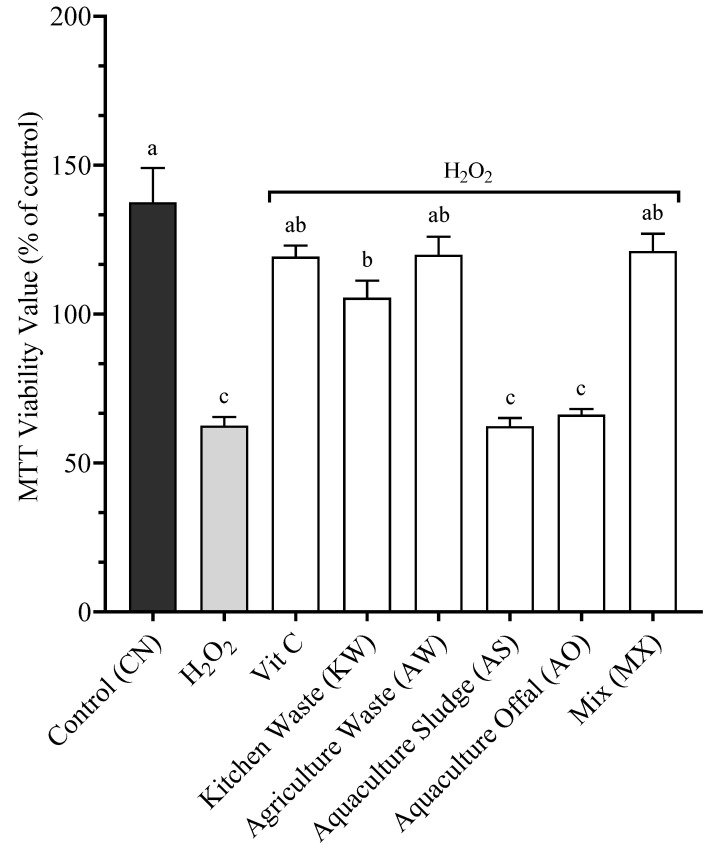
Cellular viability after 24 h of incubation with 0.1 μM H_2_O_2_ and the respective BSFL treatments included at 1.4%. Data presented are mean of n = 9 ± SEM. Different letters (a, b, c) represent statistical differences based on Tukey’s test (*p* < 0.05, one-way ANOVA).

To quantify the effects of cellular antioxidation, the cells were exposed to a fixed concentration (1.4%) of the different BSFL meals and to 0.1μM H_2_O_2_ as an oxidative agent for 24 h (Figure 1). The control (CN) received complete culture medium and 10% FBS, the negative control (H_2_O_2_) received complete culture medium, 10% FBS, and 0.1 μM H_2_O_2_, and the positive control (Vit C) received complete culture medium, 10% FBS, 0.1 μM H_2_O_2_, and 250 ppm of Vitamin C (recommended commercial feed inclusion rate, DSM). SHK-1 cells exposed to MX (121.1% ± 5.9), AW (119.9% ± 6.1), and Vit C (119.3% ± 3.8) did not have significantly different results from the Control (137.5% ± 11.6) (*p* > 0.05). Cells exposed to KW (105.5% ± 5.7) were significantly different from CN, H_2_O_2_, AS, and AO (*p* < 0.05), but not significantly different from MX, AW, and Vit C (*p* > 0.05). Cells exposed to AO (66.3% ± 1.9), H_2_O_2_ (62.5% ± 3.0), and AS (62.4% ± 2.7) were not significantly different from each other (*p* > 0.05) but significantly lower compared to all other treatments (*p* < 0.05).

### 3.2. Cellular Proliferation

**Figure 2:**
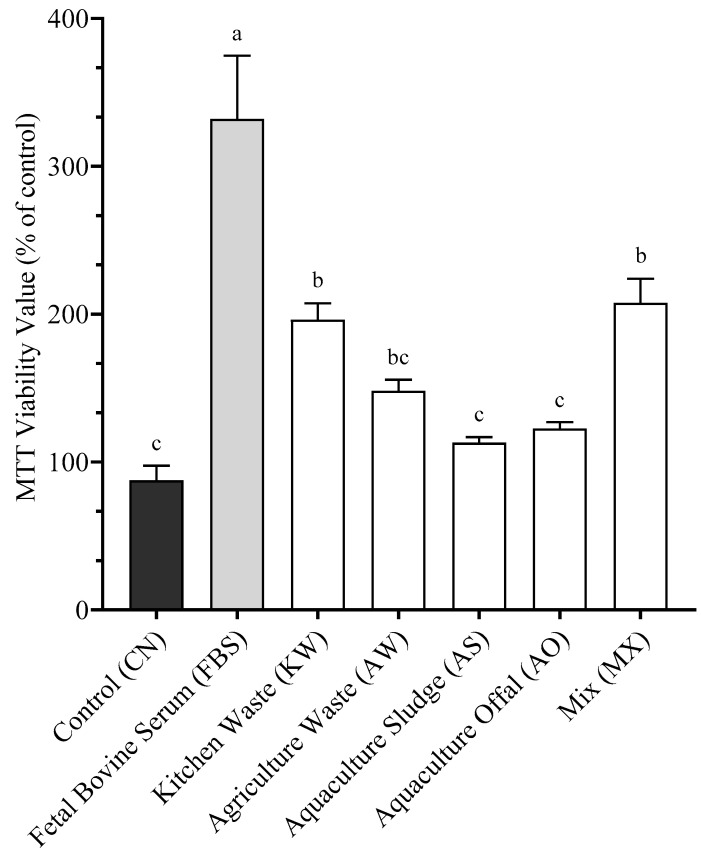
Cellular viability after 5 days of incubation with the respective BSFL meals included at 1.4%. Data presented are mean of n = 9 ± SEM. Different letters (a, b, c) represent statistical differences based on Tukey’s test (*p* < 0.05, one-way ANOVA).

To quantify the effects of cellular proliferation, SHK-1 cells were exposed to a fixed concentration (1.4%) of the different BSFLM (Figure 2) for 5 days. The control (CN) received complete culture medium and 10% FBS, and the positive control (FBS) received Fetal Bovine Serum only. FBS (332.0% ± 60.6) had the highest proliferation, significantly different from all other treatments (*p* < 0.05). MX (207.6% ± 16.3) and KW (196.3% ± 11.1) showed a similar cellular response (*p* > 0.05) to each other and to AW (148.3% ± 7.5). AW was also statistically similar (*p* > 0.05) to AO (122.6% ± 4.4), AS (113.1% ± 3.7), and CN (87.5% ± 10.1). CN, AS, and AO formed the lowest proliferation group and were significantly different from MX KW, and FBS (*p* < 0.05).

### 3.3. Protein Standardization

**Figure 3:**
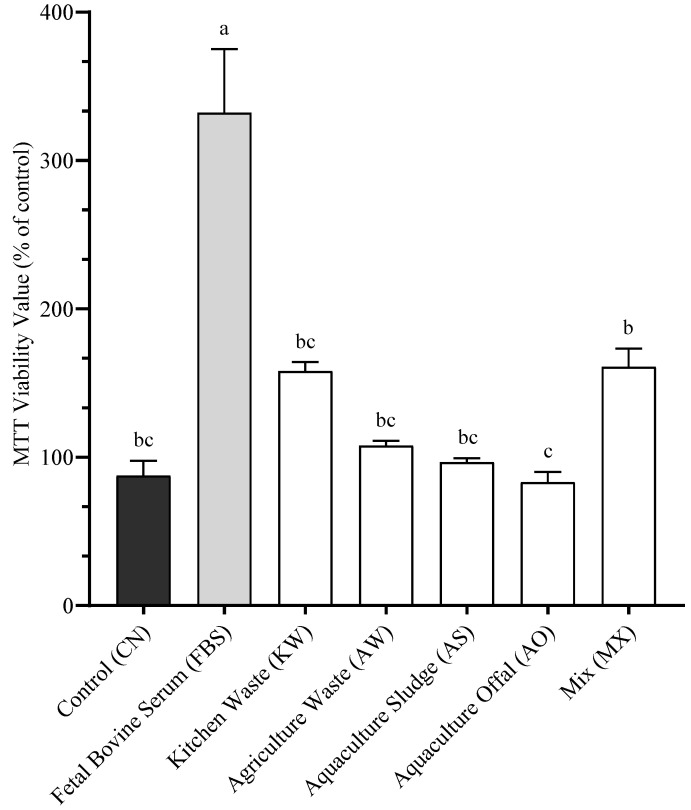
Cellular viability after 1 day of incubation with the different BSFLM included at the same protein concentration. Data presented are mean of n = 9 ± SEM. Different letters (a, b, c) represent statistical differences based on Tukey’s test (*p* < 0.05, one-way ANOVA).

To assess the effects of standardized protein dosage on cellular proliferation, SHK-1 cells were incubated for 5 days with the different BSFLM, each adjusted to the same protein concentration (1.4% M/V). The negative control (CN) consisted of complete culture medium containing 10% BSF, while the positive control (FBS) contained fetal bovine serum only. As shown in Figure 3, the FBS treatment resulted in the highest cellular viability (332% ± 42.9), which was significantly greater than all other treatments (*p* < 0.05). Among the BSFLM treatments, the MX group demonstrated the highest viability (160.75% ± 12.5), which was not significantly different from KW (158% ± 6.1), AW (107.75% ± 3.3), AS (96.63% ± 2.8), or the CN (87.5% ± 10.1). The lowest viability was observed in the AO group (83.13% ± 7.0), which was statistically similar to CN, AS, AW, and KW, but significantly lower than MX (*p* < 0.05).

### 3.4. Cellular Starvation

**Figure 4:**
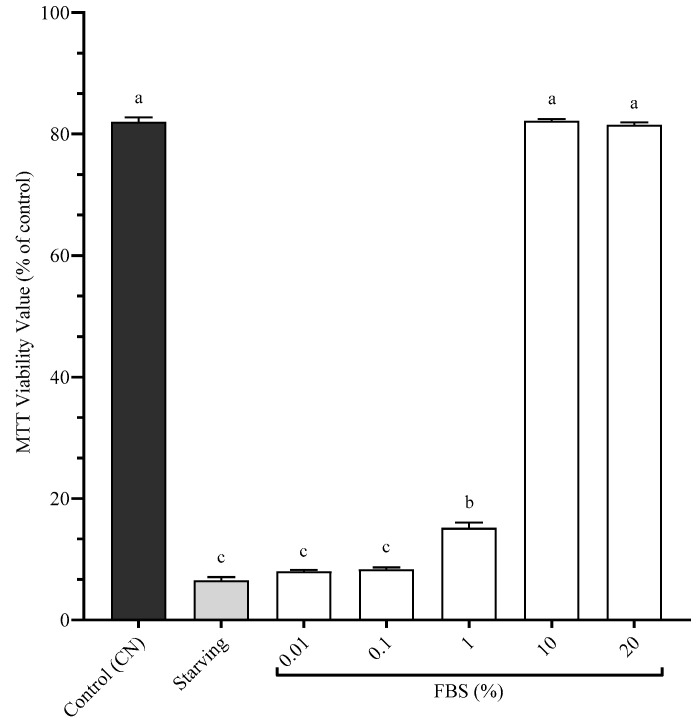
Cellular viability of SHK-1 being exposed to starvation and different doses of FBS. Data presented are mean of n = 9 ± SEM. Different letters (a, b, c) represent statistical differences based on Tukey’s test (*p* < 0.05, one-way ANOVA).

**Figure 5:**
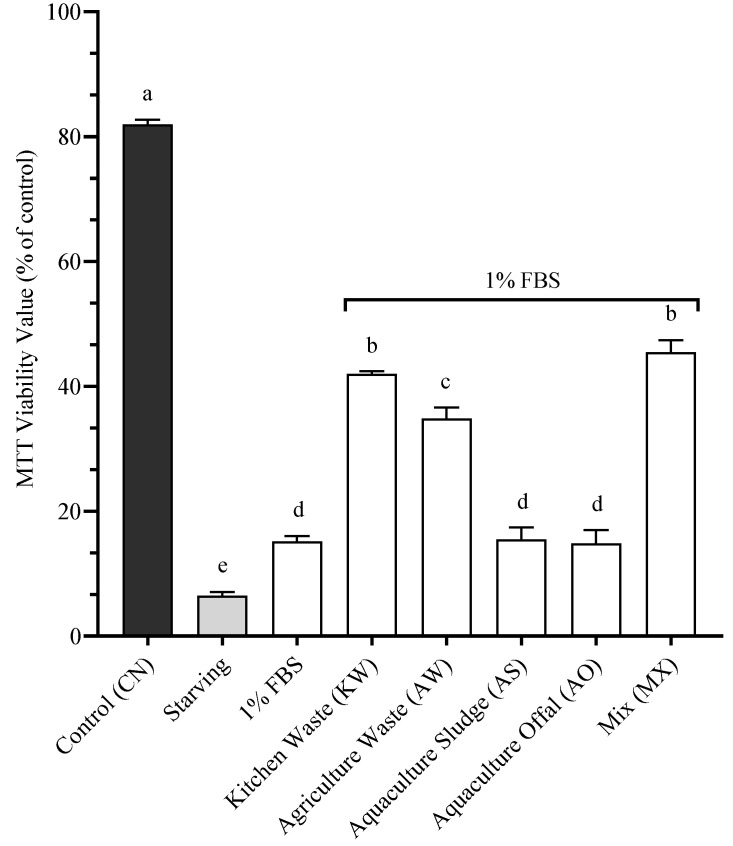
Cellular viability of SHK-1 being exposed to 1.4% inclusion of the different BSFL meals and 1% FBS following a 24 h of starvation. Data presented are mean of n = 9 ± SEM. Different letters (a, b, c, d, e) represent statistical differences based on Tukey’s test (*p* < 0.05, one-way ANOVA).

To determine the effects of our BSFL on SHK-1 post-starvation, it was first required to determine the minimum concentration of FBS that improves cellular viability after a starvation period (Figure 4). The Control (CN) received complete culture medium and 10% FBS, the Starving treatment lacked any FBS, and the other treatments received incremental doses of FBS. The treatments with 10% (82.2% ± 0.3) and 20% (81.5% ± 0.4) FBS were statistically similar to CN (82.2% ± 0.7) (*p* > 0.05). The treatment of 1% FBS (15.2% ± 0.9) was statistically different than all the other treatments (*p* > 0.05). The treatments of 0.1% (8.33% ± 0.3) and 0.01% (8.0% ± 0.3) FBS were statistically similar to the Starving treatment (6.5% ± 0.6) (*p* > 0.05). 1% FBS was determined to be the minimum concentration of FBS to significantly improve cellular viability post-starvation. Figure 5 presents the cellular viability of SHK-1 cells following a 5-day incubation period with experimental BSFL meals at 1.4% inclusion, supplemented with 1% FBS, after a 24 h starvation period. The control treatment (CN) consisted of complete culture medium containing the standard 10% FBS and showed the highest viability (82.2% ± 0.7). The Starving treatment, which contained complete medium without FBS supplementation, exhibited markedly reduced cellular viability (7.3% ± 0.5). Among the experimental treatments, MX (45.5% ± 1.9) and KW (42.0% ± 0.4) demonstrated significantly higher cellular viability than other BSFL-based meals (*p* < 0.05), yet no statistical difference was found between these two treatments (*p* > 0.05). The AW showed intermediate viability levels (34.8% ± 1.8), statistically distinct from all other treatments (*p* < 0.05). The treatments resulting in the lowest cellular viability were AS (15.5% ± 1.9), 1% FBS (15.2% ± 0.9), and AO (14.8% ± 2.2), with no significant differences among these groups (*p* > 0.05)

## 4. Discussion

### 4.1. Antioxidation

Polyphenols are well recognized plant-derived compounds with established antioxidant, anti-inflammatory, and immunomodulatory properties [32,33]. Their ability to scavenge reactive oxygen species (ROS), regulate redox-sensitive pathways, and stabilize cellular membranes makes them highly relevant for aquafeed applications [34,35,36,37]. Prior work confirmed significantly higher polyphenol concentrations in BSFLM reared on plant-based substrates compared to animal-based ones, which aligns with the superior antioxidative responses observed in the MX, AW, and KW treatments [14].

In our assay, these plant-based BSFLM treatments significantly improved SHK-1 viability under oxidative stress, performing comparably to vitamin C, a known antioxidant in aquafeeds [33]. Interestingly, the antioxidant effects of the plant-based BSFLM were observed even without added oxidative stress, suggesting a possible basal activation of endogenous defenses, potentially involving Nrf2 signaling, as shown in other studies of plant-derived antioxidants [35,38].

Conversely, animal-based treatments (AO and AS) did not confer protection, corroborating reports of lower polyphenol content in larvae fed on animal-derived waste [13,34]. In particular, the AS substrate yielded consistently poor outcomes across all assays. Previous studies have noted reduced nutritional value or bioactive potential of sludge-fed BSFL [14,39]. These negative cellular effects may reflect not only the lower nutritional value of sludge-based meals but also the potential transfer of undesirable compounds, including heavy metals, dioxins, polychlorinated biphenyls (PCBs), polybrominated diphenyl ethers (PBDEs), organochlorine pesticides (OCPs), polycyclic aromatic hydrocarbons (PAHs), and per- and polyfluoroalkyl substances (PFAS), from the aquaculture sludge into the larvae [40]. In the current research, these compounds were not quantified and thus will warrant further investigation to determine their concentration in BSFL and downstream cellular effects. That being said, there is also evidence that BSFL can contribute to the detoxification of contaminated substrates, including partial removal of heavy metals and degradation of organic pollutants [41,42]. The extent and reliability of such remediation, however, remain to be fully elucidated and should be addressed in future studies.

While this study focused specifically on polyphenols, given their well-documented effects on antioxidative responses in aquaculture species, BSFLM contains a broader spectrum of bioactive compounds that may also contribute to the observed cellular outcomes. Beyond polyphenols, compounds such as flavonoids, carotenoids, and vitamins C and E are recognized as antioxidants, and their roles in cellular protection warrant further investigation. Moreover, BSFL can bioaccumulate additional bioactives from their feed substrates, including triterpenes and phenolics with documented antioxidant properties [36]. Indeed, Rodriguez-Gonzalez et al. (2025) demonstrated that BSFLM derived from olive by-products contained triterpenes rather than phenolic precursors, underscoring the complex metabolic transformations involved in substrate-specific bioactive transfer [19]. In addition, several other classes of bioactives have been identified in BSFL. Chitin, a structural polysaccharide of the larval exoskeleton, exhibits antimicrobial properties, while lauric acid has demonstrated antiviral and antibacterial activity [43,44]. BSFL are also rich in antimicrobial and antioxidant peptides, which can inhibit pathogenic microorganisms and scavenge free radicals [44]. Novel polysaccharides isolated from BSFL have shown immunomodulatory activity through activation of the host innate immune system [45]. Furthermore, BSFL are capable of accumulating essential vitamins from their substrates, such as vitamin E, which enhances antioxidative potential when larvae are reared on seaweed or vegetable-based diets, and vitamins B1, B2, and C, which contribute to fundamental metabolic processes [46,47]. Together, these findings emphasize that the functional value of BSFLM extends well beyond polyphenols, and future studies should systematically assess the contribution of these additional bioactives in shaping antioxidative, immunomodulatory, and proliferative responses.

### 4.2. Proliferation

Cellular proliferation is influenced by both nutrient availability and cellular metabolic readiness [48,49]. In our assays, BSFLM derived from KW and MX diets significantly enhanced SHK-1 proliferation compared to AO and AS. While polyphenols may have contributed to these effects through their antioxidative and cytoprotective properties, differences in nutrient composition also appear relevant. Among the treatments, KW contained the highest concentrations of indispensable amino acids, including arginine, leucine, isoleucine, and valine, alongside elevated levels of glutamic acid, aspartic acid, and serine. These amino acids are essential for protein synthesis, nucleotide biosynthesis, and mitochondrial metabolism, all of which support proliferative activity [50,51]. By contrast, MX did not contain the highest amino acid levels but did provide a balanced profile combined with high polyphenol concentrations and beneficial fatty acids such as oleic and lauric acid. These compounds are known to contribute to membrane stability, cellular energy metabolism, and oxidative protection [52,53,54]. Thus, the enhanced proliferation observed in KW likely reflects its superior amino acid content, while the positive effects of MX may derive from the synergistic contribution of multiple nutrient classes. Collectively, these results highlight that both amino acid availability and bioactive compounds from plant-based substrates can shape the bio-functional properties of BSFLM, and that substrate-driven differences can translate into distinct cellular outcomes.

### 4.3. Proliferation (Standardized Protein)

Even when BSFLM treatments were standardized to equal protein concentrations, MX and KW continued to yield higher viability than animal-based diets. This supports the hypothesis that functional effects are not driven solely by protein but also by substrate-derived bioactives such as polyphenols and beneficial lipids [14,55].

These findings reflect growing evidence that the bioactivity of insect meals depends on their micronutrient and phytochemical content. Components like chitin, antimicrobial peptides, and specific fatty acids may all contribute to immunomodulatory and metabolic effects [56,57]. Moreover, polyphenols have been shown to influence mitochondrial function by enhancing biogenesis (PGC-1α, TFAM, NRF-1), protecting against oxidative stress via Nrf2, and regulating mitochondrial dynamics [58,59,60]. This may explain why cell viability remained elevated even when protein levels were equalized.

### 4.4. Starvation

The starvation assay provided further evidence of the functional potential of plant-based BSFLM. Once 1% FBS was confirmed as the minimum dose for cell recovery, MX and KW treatments significantly improved viability beyond FBS alone. This suggests that their bioactive content supported metabolic resilience under nutrient deprivation, conditions common in aquaculture during fasting, weaning, or stress.

Starvation is well known to disrupt redox homeostasis, as the depletion of dietary antioxidants limits the ability of cells to counteract accumulating ROS, leading to oxidative damage and impaired cellular function [61,62]. While compensatory upregulation of endogenous antioxidant enzymes such as SOD, CAT, and GPx occurs during starvation, these defenses are often insufficient to fully prevent lipid peroxidation and oxidative injury [63]. In this context, the provision of exogenous antioxidant compounds is critical. Polyphenols abundant in MX and KW treatments may have supplied additional antioxidant capacity, directly scavenging ROS and supporting mitochondrial stability.

Polyphenols have been shown to activate AMPK, a key energy sensor, promoting catabolic pathways and mitochondrial biogenesis under energy-limited states [60,64,65]. Additionally, they enhance autophagy, recycling cellular components to maintain energy balance and homeostasis [66]. These mechanisms align with the increased SHK-1 viability observed in MX and KW treatments after nutrient stress. Such cytoprotective effects are especially relevant for aquaculture species facing transient nutritional gaps or environmental challenges.

### 4.5. Relevance of Oxidative Stress in Aquaculture

Oxidative stress is a pervasive challenge in fish farming, arising from multiple stressors including crowding, handling, transport, water quality fluctuations, and pathogen exposure. For instance, high stocking densities have been shown to elevate cortisol and upregulate antioxidant enzyme activity such as superoxide dismutase (SOD) and catalase in tilapia, reflecting the strong link between fish immune response and antioxidant capacity [67]. Transport stress also elevates cortisol and induces oxidative stress markers, with intestinal oxidative products such as 4-hydroxynonenal (HNE) responding markedly to handling [68]. Similarly, abiotic stressors such as fluctuations in water temperature, salinity, pH, and dissolved oxygen have been demonstrated to disrupt redox balance and impair oxidative health status in aquaculture species, underscoring their major role in survival and productivity [69]. In addition, biotic stressors such as mycotoxins or viral infections exacerbate oxidative stress by downregulating Nrf2 signaling, reducing antioxidant enzyme activity, and promoting ROS accumulation, thereby facilitating pathogen replication [70]. Functional dietary additives have been identified as promising interventions to mitigate such stress, supporting fish resilience by modulating neurotransmission, energy metabolism, and immune defenses [71]. The ability of BSFLM to maintain cellular function under oxidative and nutrient stress therefore positions it as a promising functional ingredient for aquafeeds targeting resilience and recovery in practical aquaculture settings.

Our in vitro observations that BSFLM derived from plant-based substrates enhanced cellular antioxidant defenses are consistent with organism-level studies in fish and shrimp, where dietary inclusion of BSFLM has been shown to modulate antioxidant capacity and immune function. For instance, Senlin et al. (2017) demonstrated that dietary defatted BSFLM enhanced antioxidant status in Jian carp, supporting the hypothesis that insect meals can strengthen redox balance in aquaculture species [72]. Similarly, in African catfish, Fawole et al. (2020) reported increased catalase activity, reduced superoxide dismutase (SOD), and numerically lower malondialdehyde (MDA) concentrations, indicative of improved antioxidant potential and immunostimulation when BSFLM was included in diets [73]. Evidence from crustaceans reinforces this trend as well as He et al. (2022) showed that Pacific white shrimp fed BSFLM diets exhibited significantly higher activities of antioxidant enzymes in serum, particularly through the SOD–CAT–GSH-Px cascade, which provides the first line of defense against reactive oxygen species [74]. More recently, Abd-El Gawad et al. (2025) confirmed that dietary replacement with defatted BSFLM in tilapia enhanced antioxidative indices without inducing inflammation [75]. Notably, total antioxidant capacity, SOD, CAT, and glutathione peroxidase all increased in liver and spleen tissues, with clear benefits observed during bacterial challenge trials.

Taken together, these organism-level studies support our cellular results, indicating that the antioxidant potential of BSFLM is not only observable in vitro but can also translate into improved resilience and immune function in aquaculture species. Importantly, the consistent observation across different species suggests that the substrate-driven modulation of BSFLM composition may have broad implications for enhancing oxidative stress defenses at both the cellular and whole-organism levels.

This study provides novel insights into the cellular effects of BSFLM; however, several limitations should be acknowledged. First, the use of an in vitro SHK-1 salmonid cell line, while valuable for mechanistic screening, cannot fully replicate the systemic, immune, and metabolic interactions present in whole organisms, thus requiring validation through in vivo feeding trials. Second, only five substrate types were tested, and their composition may vary seasonally or across production sites, limiting broader generalizability. Finally, although proximate composition, amino acids, fatty acids, and polyphenols were quantified, other bioactive compounds such as flavonoids, carotenoids, vitamins, antimicrobial peptides, and chitin derivatives were not analyzed, making it difficult to attribute functional effects to specific molecules.

Several next steps are needed to strengthen and expand upon the present findings. First, broaden the characterization of bioactive compounds to include not only polyphenols but also carotenoids, vitamins, peptides, and chitin derivatives, providing a more complete picture of the mechanisms behind antioxidative and proliferative effects. Second, evaluate whether common feed manufacturing processes (e.g., drying, extrusion, pelleting) alter the antioxidant capacity of polyphenol-rich BSFL meal. Third, conduct dose–response in vivo studies to determine the minimum inclusion level of BSFL meal that enhances antioxidative responses without substantially increasing feed cost. Finally, perform a comparative assessment of in vitro and in vivo antioxidative responses to identify potential correlations between the two models. Together, these next steps will help clarify the full functional potential of BSFLM and guide its targeted use as a high-value functional ingredient in sustainable aquafeeds.

## 5. Conclusions

This study provides the first cellular-level evidence that BSFLM, particularly when derived from polyphenol-rich plant-based diets, offers significant functional benefits for aquaculture applications. Considering that the protein or lipid contribution of BSFLM might not be major due to its high cost and lower inclusion level, this novel feed component can be utilized more as a functional ingredient in aquafeeds. Using the SHK-1 cell line, we demonstrated that BSFLM can enhance antioxidative defense and support cellular proliferation under both nutrient-rich and nutrient-deficient conditions. Notably, these effects were independent of protein content, underscoring the importance of polyphenol bioactivity in determining functional outcomes. The SHK-1 cell model proved to be a sensitive and cost-effective platform for evaluating bio-functional feed ingredients, offering valuable insights prior to in vivo validation. Our findings support the strategic use of BSFLM as a high-value functional ingredient or additive in aquafeed formulations, encouraging further research into the optimization of insect diets to enhance specific bioactive properties and supporting the targeted design of next-generation functional aquafeeds.

## Data Availability

Dataset available from the authors on request.

## Abbreviations

The following abbreviations are used in this manuscript:

AO	Aquaculture Offal (diet)
AS	Aquaculture Sludge (diet)
AW	Agricultural Waste (diet)
BSFL	Black Soldier Fly Larvae
BSFLM	Black Soldier Fly Larvae Meal
CN	Control (complete medium + 10% FBS + 1% antibiotic/antimycotic)
FBS	Fetal Bovine Serum
KW	Kitchen Waste (diet)
MTT	[3-(4,5-Dimethylthiazol-2-yl)-2,5-diphenyltetrazolium bromide] (assay)
MX	Mixed Waste (diet)
ROS	Reactive Oxygen Species
SHK-1	Salmon Head Kidney—1 (cell line from *Salmo salar*)
Vit C	Vitamin C (positive control in antioxidation assay)
